# The Significance of Angiopoietin Valency in Vascular Health and Disease

**DOI:** 10.3390/cells15090820

**Published:** 2026-04-30

**Authors:** Yan Ting Zhao, Devon D. Ehnes, Julie Mathieu, Hannele Ruohola-Baker

**Affiliations:** 1 Institute for Stem Cell Research and Regenerative Medicine, University of Washington, Seattle, WA 98109, USA; ytz@uw.edu (Y.T.Z.); dehnes@uw.edu (D.D.E.); jmathieu@uw.edu (J.M.); 2 Biochemistry Department, School of Medicine, University of Washington, Seattle, WA 98195, USA; 3 Comparative Medicine Department, University of Washington, Seattle, WA 98195, USA

**Keywords:** protein design, angiogenesis, Tie2, angiopoietins, blood vessel

## Abstract

**Highlights:**

**What are the main findings?**

- F-domain valency determines the activity of Ang 1 and Ang 2 in the Tie2 pathway;
- Synthetically designed proteins precisely control Tie2 activity;
- F-domain valency of four or fewer is Ang 2-like, whereas six or more demonstrates Ang 1-like activity.

**What are the implications of the main findings?**

- Angiopoietins and Tie2 signaling play a critical role in regulating vascular health.
- Well-defined ligand valency enables precise control of Tie2 signaling for treating vascular diseases.

**Abstract:**

The Angiopoietin–Tie2 pathway is a key regulator of postnatal vascular maintenance and remodeling, regulating vascular barrier function and integrity. While the opposing roles of the ligands Angiopoietin-1 (Ang 1) and Angiopoietin-2 (Ang 2) have been recognized for decades, the structural mechanism governing their distinct signaling outputs has only recently been elucidated. As artificial intelligence and protein design continue to develop, emerging evidence suggests that ligand valency and receptor clustering are key determinants of Tie2 pathway activation and endothelial cell function; that is, “form follows function”. This review summarizes the latest discovery in the structural biology and signaling mechanism of the Tie2 pathway using protein design to decode the ligand–receptor interactions. Probing the underlying molecular basis of Tie2 offers new therapeutic opportunities for targeting diseases, featuring vascular dysfunctions such as sepsis, traumatic brain injury, acute respiratory diseases, chronic inflammation, and cancer. This also highlights the next generation of AI-designed protein therapeutics.

## 1. Introduction: The Angiopoietin–Tie Pathway in Vascular Development and Diseases

The angiopoietin–Tie signaling axis is governed by a complex interplay between two tyrosine kinase receptors, Tie1 and Tie2, and their ligands, Angiopoietin-1 (Ang 1), Angiopoietin-2 (Ang 2), and Angiopoietin-4 (Ang 4) [1,2]. Tie2 (TEK) serves as the primary signal transducer. The Angiopoietin–Tie2 (Ang-Tie2) pathway is vital for vascular homeostasis and remodeling in both normal and pathological conditions. In vivo studies underscore the criticality of this axis: homozygous knockout (KO) of Tie1, Tie2, or Ang 1 in mice results in early embryonic death on E13.5, E9.5–E10.5, or E12.5, respectively, along with varying degrees of microvascular hemorrhage, edema, ruptures, and endocardial defects [3,4,5]. Notably, Tie1 KO leads to smaller hearts, while Tie2/Ang 1 KO leads to reduced vascular branching, dilated vessels, rounded endothelial cells, and a lack of perivascular cells [1,4,5,6]. Mice with a homozygous knockout of Ang 2 were able to survive up to day 14 postnatal with major defects in lymphangiogenesis, causing lymphatic edema, ischemia-induced neovascularization, and abnormal outgrowth of retinal capillaries [6,7,8]. Ang 2 KO in C57BL/6 mice were reported to live up to adulthood but exhibited poor lymphatic maturation and defects in collecting vessel phenotypes—the connection point between lymphatic and blood vessel circulation [8]. These animal studies highlight the indispensable role of the Angiopoietin–Tie2 pathway in orchestrating both embryonic vascular development and postnatal angiogenesis.

Tie2 is highly expressed in vascular and lymphatic endothelial cells during early development, one of the key pathways that regulate endothelial cell fate and function [1]. Ang 1 is continuously secreted by pericytes or smooth muscle cells that bind Tie2 for vascular maturation, stability, and barrier integrity under homeostatic conditions or in newly formed blood vessels [9,10,11]. Ang 2 is expressed and stored in Weibel–Palade bodies in endothelial cells, and it is released in response to inflammation. Ang 2 binding to Tie2 triggers the increase in vascular permeability and the expression of ICAM and VCAM on the endothelium, facilitating leukocyte adhesion and infiltration [12]. Tie2 expression can also be found in non-vascular cell lines such as hematopoietic stem cells and macrophages (Tie2-expressing macrophages, TEMs), suggesting its potential role in regulating blood cell differentiation and inflammation [13,14,15,16]. Furthermore, the Drosophila homolog of Tie2, Tie, is expressed in both germline stem cells (GSC) and intestinal stem cells (ISC), and functions to protect these essential adult stem cells against apoptosis, a process activated by the secretion of a Tie2 ligand from dying daughter cells [17]. GSC protection is critical for regenerating the germ line after a harmful insult. Microglia in the central nervous system upregulate Tie2 in response to acute injury [18].

The Tie2 pathway serves as a regulator across vascular biology, immunology, and pathology. Given its diverse roles, there is a critical need to analyze and target Tie2 to develop effective therapies. Misregulation of Tie2 signaling is observed in both acute conditions—such as sepsis [19,20], traumatic brain injury (TBI) [18,21,22,23], and acute respiratory distress syndrome (ARDS) [24,25,26,27]—and chronic diseases, including diabetes, chronic kidney disease (CKD [28,29,30,31,32]), and atherosclerosis [33,34,35,36]. Furthermore, Tie2 signaling impacts tumor angiogenesis and metastatic progression. Collectively, Tie2 signaling significantly affects disease manifestation and patient outcomes. This review explores the molecular criteria for Tie2 activation, focusing on how ligand valency influences signaling through designed proteins and their therapeutic utility in vascular disease management.

## 2. Angiopoietin–Tie2 Pathway: Structure and Signaling

Mechanistically, angiopoietins bind and cluster Tie2 receptors, triggering auto-phosphorylation of tyrosine residues in the cytosolic kinase domains [37,38,39,40,41]. These phosphorylations trigger the activation of downstream effectors (pAKT, pERK1/2, pFAK, etc.), driving endothelial cell proliferation, migration, sprouting, and immune responses, modulating angiogenesis, vascular stability, and permeability [42,43,44,45,46,47,48,49,50,51]. Ang 1 activates Tie2 and downstream pAKT, pERK1/2, and pFAK, resulting in endothelial survival, tube formation, and vascular stability. Although Tie2 expression in sprouting endothelial cells is low [52], it is suggested that Ang 1 can induce endothelial cell sprouting via FAK phosphorylation [42,53]. Under a physiological environment, endothelial cell sprouting is driven primarily by VEGF signaling rather than Tie2.

However, the role of Ang 2 in vascular endothelial cells is variable; Ang 2 is an agonist in the absence of Ang 1. Interestingly, Ang 2 can also compete with Ang 1 and inhibits pTie2 and pAKT [54,55]. In lymphatic endothelial cells, Ang 2 is a strong agonist that activates the Tie2/pAKT axis and regulates lymphatic vessel development [56,57]. The dynamic activity of Ang 2 highlights the crucial role of Ang 2 in blood and lymphatic vessel development and function. There is no doubt that significant knowledge gaps remain regarding the precise structural mechanism of the “Ang 2 switch”—specifically, how the same binding interface can toggle between agonism and antagonism, a phenomenon increasingly attributed to differences in ligand valency rather than affinity [47]. The solution hinges on the idea that “form follows function”, guided by the Modernist axiom. To investigate how valency regulates ligand–receptor interaction, we must understand the structure of the ligands and Tie2 receptors.

**Figure 1:**
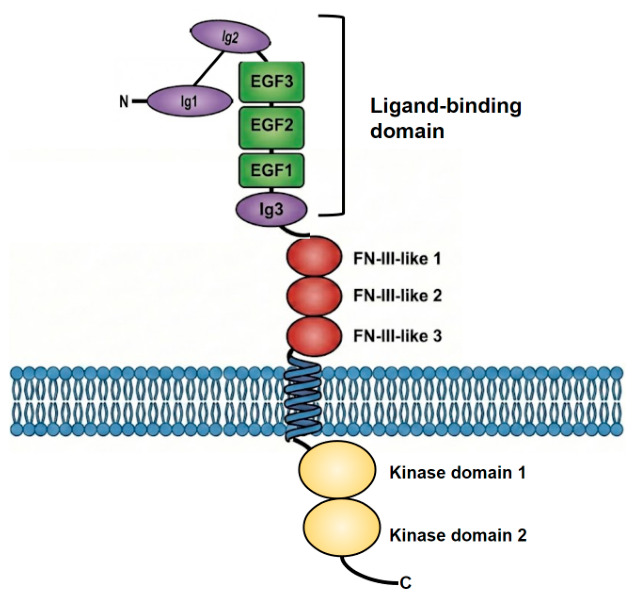
Schematic of Tie1 and Tie2 receptor structures generated by Nano Banana Pro (Gemini 3 Pro model) with modifications. The two receptors shared similar major domains and structures.

The Tie2 receptor consists of four major domains: the ligand-binding domain (LBD), fibronectin-like (FN) domain, transmembrane domain, and tyrosine kinase domain. The LBD comprises three Ig-like domains (Ig1, Ig2, and Ig3) and three EGF-like domains [58] (Figure 1). Below the LBD are three FN-III-like domains, then a transmembrane domain, followed by two cytosolic tyrosine kinase domains. Upon clustering, Tie2 cross-phosphorylate leading to the downstream activation of second messengers and transcription factors. Thus, ligand valency plays a determining role in the size and geometry of Tie2 clustering, influencing the downstream phosphorylations and activity [1].

**Figure 2:**
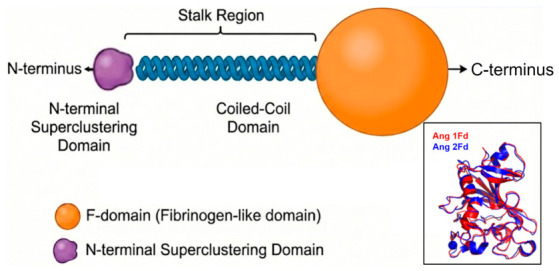
Schematic of angiopoietin 1 and 2 structures generated by Nano Banana Pro (Gemini 3 Pro model), demonstrating the structural similarity between the two ligands. Box: Super-imposed crystal structure of Ang 1 F-domain (4K0V, Chain B) [47] and Ang 2 F-domain (2GY7, Chain A) [58]. Crystal structures are produced by PyMol.

The angiopoietin ligands, Ang 1 and Ang 2, share significant structural homology but elicit opposing signaling and vascular outcomes. Structurally, Ang 1 and Ang 2 share 60% amino acid sequence homology and possess identical domain architectures: a super clustering domain (SCD), a coiled-coil domain (CCD), and a fibrinogen-like receptor-binding domain (F-domain) [47] (Figure 2). The F-domains of Ang 1 and Ang 2 exhibit nearly identical tertiary structures and comparable affinities for Tie2 binding (KD ~3 nM) [54] (Figure 2).

**Figure 3:**
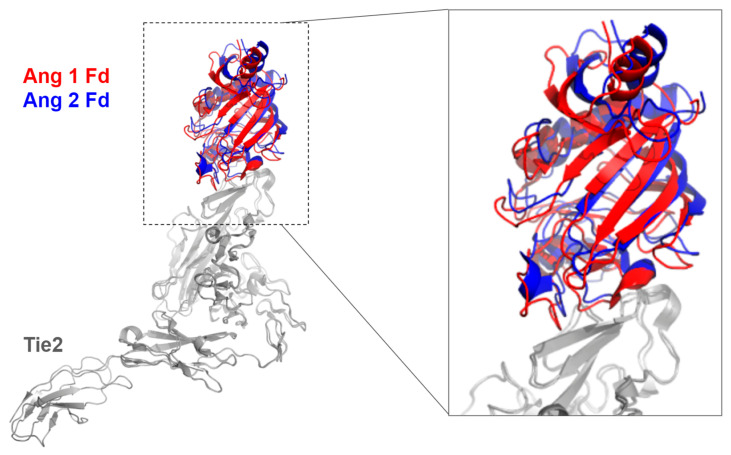
This image displays the crystal structures of Angiopoietin-1-Tie2 (Ang 1, PDB ID: 4K0V [47]) and Angiopoietin-2-Tie2 (Ang 2, PDB ID: 2GY7 [58]) superimposed at the Tie2 ectodomain. The image is produced by PyMol.

Structural analysis of the binding interface between Ang 1-Tie2 and Ang 2-Tie2 shows both ligand binding to the Tie2 receptor at the same interface on its Ligand-Binding Domain (LBD), indicating a similar binding mode (Figure 3) [47]. However, Ang 1 and Ang 2 differ significantly in their capacity for self-association. Both ligands form higher-order multimers via disulfide bonds between cysteine residues on the SCD and CCD, but Ang 1 shows a higher propensity for oligomerization [59]. In Transmission Electron Microscopy (TEM) analysis, Ang 1 exhibits trimeric, tetrameric, and pentameric, as well as higher-order multimeric structures, whereas Ang 2 exists as trimeric, tetrameric, and pentameric structures, with rare cases of higher-order multimeric structures [59]. Thus, many speculate that Ang 1 tends to form higher-order oligomers than Ang 2, and this is likely attributable to an extra cysteine residue in the Ang 1 linker region; notably, introducing this cysteine into Ang 2 facilitates increased aggregation [59]. However, both ligands lack well-defined oligomeric states to fully distinguish the effect of Ang 1 vs. Ang 2 on Tie2, which leads to further investigation of Tie2 activation using synthetic proteins to control ligand valency.

## 3. Protein Design Can Precisely Control Ligand Valency and Signaling Outcome

Numerous efforts attempted to correlate the angiopoietin oligomeric states with Tie2 signaling outcomes using natural ligands (Ang 1/2), chimeric angiopoietins [60], modified angiopoietins [48,61,62] (CompAng 1 /2 and other variants), computationally designed protein scaffold conjugated to Ang 1 F-domain [21,63,64], Tie2 antibodies (MT100 [65], hTAAB [66]), or Ang 2 antibodies (ABTAA [45]). Firstly, engineered antibodies ABTAA against Ang 2 demonstrate that Ang 2 at high enough valency shows pTie2, pERK, and pAKT/FOXO activations, but the ligand valency is largely unknown [45]. Similarly, antibodies against Tie2 receptors (MT100 [65], hTAAB [66]) cluster and activate Tie2, but the oligomeric states of the receptor complexes remain ambiguous. The antibody approach cannot provide a precise correlation between the oligomeric state of the ligand valency or receptor clusters with downstream signaling output. Chimeric mouse angiopoietins generated by swapping the F-domains of Ang 1 and Ang 2 suggested the F-domain is the determinant for Tie2 activation, not the oligomeric state [47,60]. Nevertheless, a growing body of evidence indicates that the F-domain of Ang 1 or Ang 2 at the appropriate valency is sufficient for inducing Ang 1-like or Ang 2-like activity.

Table 1 summarizes the valency of modified angiopoietin constructs and their activity in the Tie2 pathway. Although many of them lack a well-defined valency status and exhibit conflicting results in valency–Tie2 activity correlation, they provide great insight into the role of ligand valency. The monomeric ligand (Ang 1 F-domain alone) does not activate Tie2. Most dimeric ligands do not activate Tie2, with one exception (CA1-3) that shows pTie2 and pAKT activation, but non-reducing electrophoresis shows that higher-order oligomers exist within the CA1-3. Trimeric and tetrameric ligands activate pTie2 and pAKT, with the same caveat of mixed oligomeric states. The pentameric Comp-Ang 1/2 constructs activate pTie2/pAKT. Comp-Ang 1/2 are widely used to investigate the Tie2 pathway and therapeutic applications for vascular diseases. The F-domain of Ang 1 or 2 was fused with the short coiled-coil domain of cartilage oligomeric matrix protein (COMP) to generate Comp-Ang 1/2, respectively, expected to be pentamers, and both Comp-Ang displayed Ang 1-like agonism. However, biochemical analysis shows higher-order oligomers also exist in Comp-Ang, making it difficult to correlate Tie2 activation with ligand valency. In summary, all previous work contributed significant insight to support that ligand valency plays a critical role in determining Tie2 signaling outcomes.

**Figure 4:**
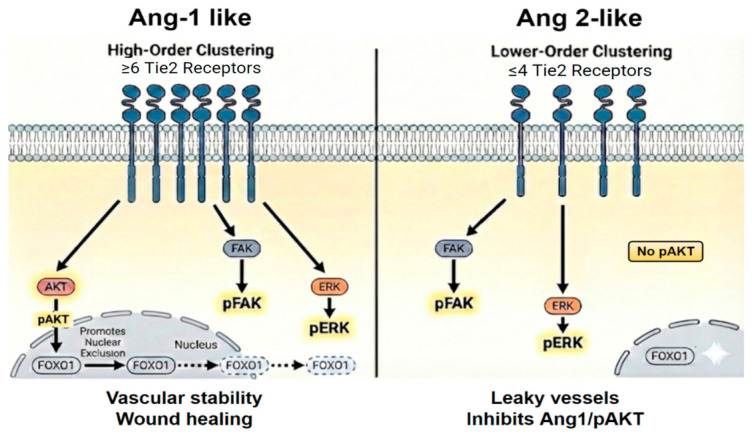
Based on the F-domain scaffolds, ligand valency determines the size of Tie2 receptor clusters, resulting in differential signaling and functional output. Ligands with valency of four or lower produce Ang 2-like activity, while ligands with valency of six or more produce Ang 1-like activity. Black solid arrows indication pathway activation. Dotted arrows indicate FOXO translation out of the nucleus. Schematic was generated using Nano Banana Pro (Gemini 3 Pro model).

The generation of ligands with well-defined valency status is needed to precisely dissect the molecular regulation of Tie2 signaling. The emergence of protein design technology enables a novel approach to precisely control the molecular interaction between receptors and ligands [67,68]. Protein design enables a rapid generation of protein scaffolds and binder libraries. The wide range of protein scaffolds can be modified to self-associate into higher-order multimers at well-defined oligomeric states. The F-domain scaffolds are a range of designed protein scaffolds conjugated with Ang 1 F-domain at a well-defined homogeneous oligomeric state [21,63,64]. Using the F-domain scaffolds, Ang 1-like vs. Ang 2-like activities were distinguishable based on defined oligomeric state (Figure 4). Low F-domain valency scaffolds—dimeric, trimeric, and tetrameric—behaved like Ang 2. Conversely, high-valency F-domain scaffolds—hexameric, octameric, tetrahedral, octahedral, and icosahedral—behaved like Ang 1 [21,63,64].

Similar to Ang 2, low-valency F-domain scaffolds (H3 and Tet1-A) were able to compete with Ang 1 and inhibit pAKT activation [21,54,55]. The F-domain scaffolds revealed the minimum molecular requirement for Ang 1-like vs. Ang 2-like Tie2 signaling output. However, current scientific understanding is still limited by the absence of a truly homogenous pentameric ligand. While COMP-based constructs and natural Angiopoietins (1 and 2) are capable of forming pentamers, they typically manifest as mixed oligomers, leading to a gap in knowledge regarding the specific signaling behavior—agonistic or antagonistic—of a pentameric valency. Leveraging artificial intelligence for protein design may provide the means to surpass existing technical hurdles in the development of a pentameric ligand.

Receptor geometry can also influence Tie2 cross-phosphorylations, co-receptor interactions, and adapter protein binding in Tie2’s cytosolic domain. F-domain scaffolds with the same valency but differing in geometry did not generate a significant difference in Tie2 activation [21]. Further investigation is needed to analyze the relationship between receptor geometry and signaling bias.

Previous studies have begun to explore the effects of ligand valency on Tie2–co-receptor interactions. Co-receptors, such as Tie1, ⍺5ꞵ1 integrin, and VE-PTP, regulate angiopoietins’ activity on Tie2. Upon Ang 1 or higher valency agonists binding, Tie2 is found to colocalize with ⍺5β1 and promote pFAK, pAkt, and pERK activation [21,51], whereas such an effect is not observed with lower-valency ligands [21]. Since Ang 1 F-domain has been shown to interact with integrin [51,69,70], F-domain likely binds to both Tie2 and integrin, forming a complex that is further stabilized and strengthened by higher valency. A de novo-designed Tie2 binder is also highly desirable as the native angiopoietins are thermally unstable, and the F-domain is also known to bind integrins [69,70] in addition to Tie2. This raises further questions regarding whether the F-domain or valency plays a more significant role in the association of Tie2 with its co-receptors.

Vascular endothelial protein tyrosine phosphatase (VE-PTP) is a Tie2 inhibitor. In vascular endothelial cells expressing vascular endothelial protein tyrosine phosphatase (VE-PTP), the role of Ang 2 is variable. Ang 2 acts as an agonist in the absence of Ang 1, but it also competes with Ang 1 and inhibits pTie2 and pAKT [54,55]. In contrast, in lymphatic endothelial cells that lack VE-PTP expression, Ang 2 acts as a consistent agonist, activating the Tie2/pAKT axis [56,57]. It is hypothesized that high-valency ligands, like Ang 1, may have induced large clusters of Tie2 that exclude VE-PTP from dephosphorylating Tie2. On the other hand, lower-valency ligands, like Ang 2, make smaller Tie2 clusters that are more susceptible to VE-PTP dephosphorylation, similar to the phase-separation process observed in T-cell receptor (TCR), in which the negative regulators are spatially excluded upon TCR activation [71,72].

Despite the structural similarity, Tie1 and Tie2 display very different signaling roles in angiogenesis. Tie1 is known as an orphan receptor that does not directly bind angiopoietins but is critical for modulating Tie2 surface presentation and signal transduction [73,74,75,76,77,78]. Using fluorescence resonance energy transfer (FRET), Ang 2 is shown to promote Tie1-Tie2 interaction, resulting in inhibitory effects in Tie2 signaling, whereas Ang 1 disrupts the Tie1-Tie2 interaction, leading to stable vessels [47,77]. These observations suggest that Ang 1 or high valency ligands are likely generating large clusters of Tie2 receptors, which excludes Tie1, whereas Ang 2 and low valency ligands make smaller Tie2 clusters, exposing more Tie2 for Tie1-Tie2 association. In contrast, other studies argue that Tie1-Tie2 heterodimer lowers the threshold for Tie2 activation, and is suggested to enable Ang 2 agonism by lowering the Tie2 activation threshold [74,76,78]. Since Ang 2 also forms higher oligomers, it is unclear whether Tie1 truly lowers Tie2 activation threshold or if it is driven by Ang 2 at higher valency. Interestingly, during inflammatory states, the Tie1 ectodomain is cleaved (shedding), an event that renders Tie2 susceptible to antagonism and contributes to vascular leakage [74,77]. It is apparent that more work is needed to delineate the regulation and function of the Tie1-Tie2 complex.

Artificial intelligence using RF diffusion has successfully generated fully synthetic de novo-designed binders [67,68]. These synthetic binders demonstrated superior affinity, specificity, and activity over the native growth factors such as fibronectin [79], FGF [80], TGF-β ligands [81], and insulin [82] (Table 2). Perhaps fully synthetic Tie1 and Tie2 binders with high specificity are needed to explore the true mode of action and function of the Tie1-Tie2 complex. Similarly, combining the Tie2 binder with the integrin binder (⍺5β1mb [79]) can also enable us to investigate the role of F-domain vs. valency in the Tie2–integrin complex. Protein design is a valuable tool that empowers biologists to not only dissect the relationship of ligand valency and Tie2 signaling output but also allows the precise manipulation of Tie2 signaling to control vascular dysfunctions in diseases.

## 4. Therapeutic Application of Design Proteins in Tie2-Mediated Vascular Dysfunction

**Figure 5:**
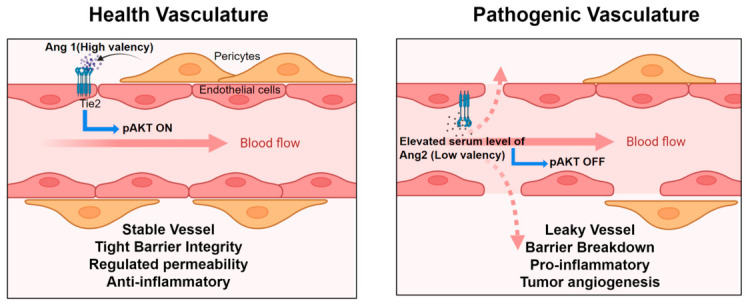
The dual roles of Tie2 signaling in healthy vascular maintenance and pathogenic vessel formation. Black arrow indicates the secretion of Ang 1 by pericytes. Pink dotted arrows indicate vascular leakage. The schematic is created with BioRender.com.

The Angiopoietin–Tie2 signaling axis is central to both healthy and diseased blood vessels (Figure 5). Specifically, angiopoietins of varying valencies are involved in regulating diverse vascular functions and disease pathology. Herein, we review the role of this pathway in acute and chronic inflammation, as well as in cancer.

### 4.1. Acute Vascular Failure (Sepsis, ARDS, and TBI)

Acute vascular failure refers to a series of severe vascular complications, including sepsis, acute respiratory distress syndrome (ARDS), and traumatic brain injury (TBI), characterized by leaky vasculature, inflammation, and hypoxia, which require immediate medical intervention to restore circulation and prevent permanent organ damage or death. These complications can be triggered by various conditions, including illnesses causing chronic or robust inflammation, and in response to infectious diseases.

Sepsis occurs when an overwhelming infection triggers systemic inflammation and widespread endothelial dysfunction. Experimental studies support a causal role for Angiopoietin/Tie2 signaling dysregulation in sepsis outcomes. Animal models of sepsis show that upregulating Tie2 signaling has a protective effect. For example, adenoviral overexpression of Ang 1 in mice with endotoxic-shock-preserved blood pressure, prevented capillary leakage, and dampened inflammation [24]. Furthermore, in animals with polymicrobiological abdominal sepsis in multiple organ dysfunction, the intravenous administration of recombinant Ang 1 improved the condition and survival time of the animals by preserving vascular barrier function [83] and reducing inflammation [84].

Conversely, reducing Tie2 activity or activating the antagonistic Ang 2 ligand worsens sepsis. Mice with even one Tie2 allele knocked out suffer greater vascular leakage and higher mortality under septic challenge [85]. Likewise, excess Ang 2 is harmful: both partial Ang 2 gene deletion [86] and lung-targeted RNAi against Ang 2 [87] have shown protective effects from vascular leak and death in septic mice. These and other studies have established hallmark Tie2 pathway signatures in sepsis in the clinical setting. The loss of Tie2 activity disrupts vascular stability, resulting in leaky capillaries, edema, leukocyte diapedesis, and microthromboses [88,89,90]. Clinically, studies have shown that an acute surge of circulating Ang 2, on the magnitude of 10–100 times over healthy individuals [91], is routinely observed early in sepsis and correlates with loss of vascular integrity and organ failure [20,92]. Correspondingly, the Ang 2/Ang 1 ratio in blood becomes markedly elevated, indicating severe Tie2 pathway impairment [19]. This Ang 2 elevation is such a consistent feature that Ang 2 has been proposed as a dynamic biomarker of sepsis severity. Interestingly, clustering Ang 2 into higher-order multimers using antibodies ABTAA produced Ang 1-like activity and ameliorated LPS-induced sepsis in mice [45].

There is some distinction between septic infection in the lungs and secondary complications from the widespread inflammation that may or may not be caused by septic infection. The latter is known as Acute Respiratory Distress Syndrome (ARDS), a severe lung injury that occurs in response to severe respiratory inflammation caused by infections like pneumonia or SARS-CoV-2, or secondary to sepsis [26,93,94]. It is characterized by severe lung swelling that causes fluid to build up in the alveoli, impairing breathing and reducing oxygen availability in the blood. In animal models, therapeutically modulating Tie2 signaling activity defends the lungs against injury from various insults: pulmonary edema and inflammation caused by endotoxin [95], hyperoxia [96], and even chemical toxins [97,98]. Lung-specific studies in sepsis found that high Ang 2 levels make pulmonary microvessels leaky, causing protein-rich edema in airspaces and impaired gas exchange [92], but ARDS has also demonstrated connections with dysregulated Tie2 signaling, demonstrating the importance of the angiopoietin/Tie2 pathway in ARDS pathogenesis as in sepsis. Clinical studies in ARDS patients show that elevated Ang 2 in plasma or lung fluid correlates with worse oxygenation and more severe respiratory failure [99]. Notably, this Tie2 pathway disruption is not specific to infection. Even in what are known as sterile lung injuries, in which severe inflammation is triggered by surgery or other direct or indirect traumatic injury as opposed to bacterial or viral infection, studies have demonstrated a higher Ang 2:Ang 1 ratio [100,101,102], consistent with Ang 2 misregulation being the harbinger of critical illness as opposed to some specific pathogen. Tie2 misregulation has also been linked to ARDS through inherent genetic variations. Studies have shown that individuals with various polymorphisms in Ang 2 that lead to higher Ang 2 secretion are at increased risk for developing ARDS following pulmonary injury [25]. Similarly, a common variant in TEK (the Tie2 gene), which results in lower baseline Tie2 expression, was found to predispose individuals to ARDS [85].

Traumatic brain injury (TBI) damages the brain function via a cascade of events from primary mechanical injury, followed by a secondary injury phase characterized by blood–brain barrier (BBB) breakdown, cerebral edema, and neuroinflammation. This disruption allows for the extravasation of blood-borne factors (e.g., fibrinogen and albumin) and immune cells into the brain parenchyma, exacerbating neuronal death and functional impairment. The Ang-Tie2 signaling axis, a critical regulator of endothelial quiescence and vascular stability, has emerged as a central determinant of BBB integrity following TBI. The Tie2 pathway appears to drive age-related differences in TBI recovery. Juvenile mice exhibit faster restoration of BBB integrity compared to adults, a phenotype that is abolished by pharmacological inhibition of Tie2, suggesting that robust Tie2 signaling is the mechanism underlying the “plasticity” of the juvenile BBB [103]. Elevated Ang 2 levels in the serum of TBI patients have also been identified as a potential biomarker for the severity of BBB disruption [104]. Ang 1 is found to rapidly decrease upon BBB injury, while Ang 2 expression increases in the cortex, hippocampus, and microvessels [105]. Activating Tie2 using the F-domain scaffold agonist, Icos1, was able to accelerate neurovascular regeneration in mice after TBI through the activation of the Tie2-pAKT pathway [21].

### 4.2. Chronic Vascular Disorders and Inflammation

Inflammation is profoundly interrelated with the Angiopoietin–Tie2 axis. In fact, Tie2 can be seen as a regulatory switch between vascular homeostasis and inflammation [106]. In uninflamed conditions, Ang 1 from pericytes and platelets constantly activates Tie2 in the endothelium, thereby keeping blood vessels tight and non-adhesive to leukocytes. However, during inflammation, endothelial cells respond by releasing stored Ang 2, which floods the local environment and competitively inhibits Ang 1, upregulating vascular permeability. This process typically remains local and controlled, but in chronic inflammatory conditions, Ang 2 is upregulated in affected tissues, leading to abnormal angiogenesis and persistent leukocyte influx [107]. Chronic inflammation occurs as part of many diseases. Here, we discuss those most profoundly connected with the vascular system.

In chronic kidney disease (CKD), numerous studies report that Ang 2 levels are chronically elevated, both in plasma and within diseased kidneys [30,108,109]. Clinically, high circulating Ang 2 (and a high Ang 2/Ang 1 ratio) in CKD patients correlates with faster progression to end-stage renal failure [29,32]. In one cohort of 319 CKD patients, those with higher Ang 2 and Ang 2/Ang 1 ratio experienced significantly greater risk of kidney function decline, suggesting Ang 2 is not just a marker but a driver of renal injury [28]. Mechanistically, excess Ang 2 promotes glomerular and vascular injury in the kidney, whereas Ang 1–Tie2 signaling is protective. In experimental models of kidney disease (obstructive nephropathy or ischemia–reperfusion injury), overexpression of Ang 1 was shown to attenuate damage: Ang 1 preserved microvascular integrity, reduced endothelial cell apoptosis, and decreased inflammation and fibrosis in the injured kidney [110,111] by dampening the endothelial secretion of chemokine CCL2, thereby curbing monocyte/macrophage infiltration into the kidney [28]. It also directly protected the endothelium from apoptotic signals induced by Ang 2 and Wnt ligands present during injury. Blocking Ang 2 had beneficial effects: administering the Ang 2-neutralizing agent L1-10 to mice with progressive CKD significantly reduced peritubular capillary loss, macrophage accumulation, and kidney fibrosis. One study found that COMP-Ang 1 significantly reduced endotoxin-induced vascular leakage in the lung, heart, and kidney, but not in the liver or intestine, demonstrating the potential of utilizing Ang 1-like ligands for treating CKD [112].

Diabetes mellitus, especially when uncontrolled, leads to chronic vascular complications in multiple organs. A unifying feature of diabetic complications is endothelial dysfunction driven by hyperglycemia and resulting inflammation, insulin resistance, and dyslipidemia [113]. Recent evidence implicates the Angiopoietin–Tie2 system as a mediator of this dysfunction. Chronic hyperglycemia itself causes an upregulation of Angiopoietin-2 in vascular cells [114]. Consequently, diabetic patients often have elevated Ang 2 levels in their circulation. For instance, Ang 2 is higher in the serum of type 2 diabetics with microvascular complications [115], and it correlates with markers of endothelial injury and inflammation. Vasculopathy refers to a secondary disease affecting blood vessels following a primary inflammation, and diabetes can cause widespread diabetic vasculopathy [116]. For example, in diabetic kidneys, hyperglycemia-induced Ang 2 elevation in glomerular endothelial cells leads to loss of the glomerular filtration barrier. High glomerular Ang 2 was found to cause pericyte-like mesangial cell dropout and endothelial fenestration, promoting albumin leakage into urine. In patients with diabetic nephropathy, plasma Ang 2 levels correlate with proteinuria severity and disease progression [28].

One of the most common diabetic vascular complications, diabetic retinopathy, exemplifies this process. In diabetes, retinal Ang 2 is markedly increased, while Ang 1 is unchanged [117]. This shift permits retinal capillaries to become permeable and prone to leakage, a key feature of diabetic macular edema. This same study found that Ang 2 also induces inflammation in the diabetic retina by promoting leukocyte adhesion and even triggering the apoptosis of astrocytes, a retinal supporting cell [117], causing their loss and thus weakening vascular support. These insights, combined with older studies showing synergistic effects of modulating both VEGFA and Ang 2 [118,119], showed reduced inflammation and better repair of existing vasculature, leading to improved vision outcomes. These have led to new, more effective therapies: notably, faricimab, a bispecific antibody that neutralizes both VEGF and Ang 2, was approved to treat diabetic macular edema [120,121]. In phase III trials, a broad population of patients who received anti-Ang 2 and VEGF treatment had improved retinal edema and vision outcomes compared to anti-VEGF alone [122]. COMP-Ang 1 protects against renal damage by preserving vascular integrity, reducing albuminuria, and decreasing glomerular hypertrophy and fibrosis [123]. In diabetic retinopathy, COMP-Ang 1 also demonstrated promising results in stabilizing vascular integrity under hyperglycemic environments in in vitro and in vivo mouse models [124,125].

Atherosclerosis is a chronic arterial disease that can occur independently of diabetes, though studies have shown that diabetes can both induce its development and accelerate deterioration [126]. A recent study definitively linked the Tie2 signaling axis to the control of the development of atherosclerosis. Their findings indicate that Tie2 signaling in the arterial endothelium is, in fact, atheroprotective: in healthy arteries, endothelial cells experience steady laminar blood flow, which maintains high Tie2 activity (via Ang 1 from supporting cells), helping to keep the endothelium anti-inflammatory. Moreover, their human genome-wide association study identified inactivating variants in the TIE2 (TEK) gene that were associated with increased risk for developing coronary artery disease. Further mechanistic work showed this variant lies in an intronic enhancer responsive to TNFα; the risk allele impairs Tie2 upregulation under inflammatory stress, potentially making arteries more vulnerable to inflammation. In conjunction with genetic risk factors associated with atherosclerosis, this and other recent studies have found that mechanical factors inherent to macrovascular complications can further exacerbate development and progression. Regions of disturbed flow (such as branch points predisposed to plaque) have reduced Tie2 signaling and higher Ang 2, contributing to local endothelial activation [34,35,127]. In experimental atherosclerosis models, endothelial-specific deletion of Tie2 accelerates plaque formation. Mice lacking Tie2 in arterial endothelial cells developed larger atherosclerotic lesions with increased endothelial NF-κB activation, upregulated adhesion molecules VCAM1 and ICAM1, and greater infiltration of monocytes and T cells into the artery wall [35]. Loss of Tie2 was associated with increased nuclear FOXO1 and a pro-inflammatory gene expression profile in both endothelial cells and even in neighboring fibroblasts, enhancing the inflammatory state. By contrast, mice with intact Tie2 signaling had relative resistance to atherosclerosis, even when challenged with a high-fat diet and other risk factors. Collectively, these findings highlight the Tie2 axis as a major target of novel therapeutics for chronic vascular diseases and inflammation.

### 4.3. Oncogenesis and the Tumor Microenvironment

The tumor microenvironment is composed of a diverse mix of typically non-malignant cell types that are leveraged together to facilitate tumor survival and progression, including endothelial cells that will enable the growth of new blood vessels for tumor growth [128]. Accordingly, numerous antiangiogenic therapeutics have been developed to be used in conjunction with other therapeutics to help reduce tumor growth while targeting other tumor cells [129]. However, these therapeutics, which typically target the VEGF, FGF, or PDGF pathway, have shown limitations in efficacy [130]. For instance, small-molecule or antibody inhibitors against the VEGF pathway have an average life expectancy of 4–5 months, along with serious side effects, drug resistance, and cancer relapse [129,130].

Unsurprisingly, the Angiopoietin–Tie2 axis has been identified as pivotal in cancer, particularly in tumor angiogenesis, tumor inflammation, and metastasis. High Ang 2 levels have been demonstrated in many cancers, including glioblastoma [131], colorectal [132], and breast cancer [133]. These elevated levels correlate with increased microvessel density and poor prognosis, linking Ang 2 to tumor vascularization and metastatic potential [36]. Studies have found that although Ang 2 is only mildly expressed in healthy endothelial cells, tumor-associated endothelial cells often overexpress Ang 2, leading to leaky vascular integrity, hypoxic tumor environment, and upregulation of VEGF overexpression to promote vascular sprouting, leading to aberrant pathological angiogenesis [12,134]. Early studies found that using anti-Ang 2 antibodies (ABTAA) and peptide body (AMG 386) inhibits angiogenesis and tumor growth [135,136]. Accordingly, researchers have developed therapies that synergize the inhibition of VEGF and the Tie2 signaling axis, which have yielded more successful control of tumor angiogenesis in preclinical mouse models [135,137,138,139,140,141]. Elevated Ang1 levels are also associated with poor prognosis and increased tumor growth in certain types of cancers, including triple-negative breast cancer (TNBC) and gliomas [142,143].

Tie2 signaling also influences various cell types in the tumor microenvironment in addition to endothelial cells. In oral squamous cell carcinomas (OSCCs), high Ang 1/Tie2 expression in cancer cells drives epithelial–mesenchymal transition (EMT) and metastasis [144]. Tie2-expressing macrophages (TEMs) are a subset of tumor-associated macrophages that express the Tie2 receptor and are highly pro-angiogenic [145]. Ang 1 can induce p38 and Erk1/2 phosphorylation in TEMs and drive macrophage differentiation toward a pro-inflammatory state (M1) [146]. M1 macrophages are activated to eradicate pathogens and cancerous cells [147]. In contrast, Ang 2 from tumor vessels can recruit and activate these TEMs, which will further stimulate tumor angiogenesis [148]. Several groups have hypothesized that these cells are responsible for repairing damaged tumor vasculature and facilitating tumor relapse [14,149,150,151], but knocking out Tie2 in these specific monocytes did not alter the relapse rate of solid tumors [152]. One recent study even found that TEMs promoted angiogenesis in chronically ischemic brain tissue [23], supporting the studies showing that these cells can be pro-angiogenic in cancer microenvironments, which are also inherently ischemic. One study found that Ang 2 blockade did not inhibit the recruitment of MRC1(+) TIE2-expressing macrophages but impeded their upregulation of Tie2, their association with blood vessels, and their ability to restore angiogenesis in tumors [153]. Another found that impairing the ability for TEMs and other tumor cells to communicate prevented metastasis in breast carcinomas [154]. Taken together, Tie2 plays a different role in cancer. Tie2 can exhibit pro-tumor or anti-tumor characteristics in different cancers. Thus, targeting Tie2 using agonists or antagonists should be tailored to the specific cancer type.

## 5. Conclusions

The complex signaling outcomes of the Angiopoietin–Tie2 axis are fundamentally governed by ligand valency and the resulting receptor clustering dynamics, rather than simple binding affinity. Historically, Ang 1 is known as the agonist while Ang 2 is the antagonist, but this claim is obscured by the “Ang 2 switch” mechanism, better known as the context-dependent agonist/antagonist. The technological advance in protein design enables us to dissect the Ang 1 vs. Ang 2 activity based on ligand valency. Studies utilizing Comp-Ang, antibodies, angioprotein surrogates, and modified angiopoetins provided pivotal insights about Tie2 signaling and vascular outcomes. The development of F-domain scaffolds provides additional clarification about how angiopoietin valency correlates with Tie2 signaling outcome by precisely controlling ligand valency and geometry. F-domain scaffolds have demonstrated that high-order multimers (valency of 6 or more) are required to drive Ang 1-like activity, whereas low-valency ligands (valency of four or lower) mimic Ang 2-like activity. This technological and mechanistic breakthrough translates directly into clinical potential for managing vascular dysfunction.

The ability to engineer synthetic Tie2 binders offers a powerful toolkit for a spectrum of devastating conditions. By enforcing receptor clustering at the right valency and geometry, Tie2 has the potential to control acute vascular integrity crises (sepsis, ARDS, and TBI) and chronic inflammatory states (diabetic retinopathy, atherosclerosis, and CKD) more precisely. In acute and chronic vascular injuries, where the Tie2 pathway is suppressed by elevated Ang 2, high-valency agonists, F-domain scaffold Icos1 and Comp-Ang 1, offer a strategy to rescue endothelial barrier function and prevent leakage. The Tie2 pathway plays a dynamic and critical role in pathogenic angiogenesis and Tie2-expressing macrophage (TEM) immune response in different cancers. In the tumor microenvironment, where Ang 2-mediated instability facilitates aberrant angiogenesis and metastasis, targeting this axis with precise Ang 1-like super agonists remain a critical therapeutic avenue. Ultimately, the ability to control Tie2 signaling outputs through precise valency engineering represents a paradigm shift from non-specific, broad-spectrum modulation of angiopoietins to targeted vascular management.

Artificial intelligence using RF diffusion sheds light on replacing native ligands with fully synthetic, de novo-designed binders [67,68]. Many of these de novo-designed protein binders have already demonstrated superior affinity, specificity, and activity over the native growth factors. Producing native proteins at scale is challenging due to their thermal instability and high cost. Furthermore, their non-specific binding to multiple targets complicates research and therapeutic development. A de novo-designed Tie2 binder is highly desirable as the native angiopoietins and F-domain-based constructs have the same limitations. The Ang 1 F-domain is also known to bind integrins in addition to Tie2 [69,70], which can generate off-target effects when used in clinical applications. Developing completely synthetic Tie2 agonists and antagonists with precisely defined valency and novel binder scaffolds is a critical area for future research. Ultimately, the integration of structural biology and artificial intelligence in protein design marks a new era in vascular medicine. Before adapting synthetic proteins for therapeutic application, it is important to evaluate and overcome translational challenges, such as in vivo pharmacokinetics, pharmacodynamics, immunogenicity of non-native proteins, tissue-specific delivery, and toxicity. Future research must focus on elucidating the exact dynamics of Tie2 co-receptors and translating a de novo-designed, highly stable Tie2 binder at the appropriate valency into clinical trials. By mastering the valency and geometric requirements of Tie2 activation, we move closer to resolving the root causes of severe vascular dysfunctions.

## Figures and Tables

**Table 1:** Summary of ligand valency and signaling output. This stable summarizes constructs at different valencies, signaling outcome, and observed oligomeric states (homogeneous vs. heterogeneous). Refer to the List of Abbreviations for the constructs’ description.

Valency	Constructs	Signaling Activity	Observed Oligomeric State
1	F-domain (Fd)	no Tie2 activity [21,64]	Homogeneous
2	Ang 1-Fc	no Tie2 activity [63]	Homogeneous
	CA1-3	pTie2 and pAKT [62]	Heterogeneous
	GCN4-Ang 1	No Tie2 activity [61]	Heterogeneous
	Fc-A1	no Tie2 activity [62]	Heterogeneous
3	GCN4-Ang2	no Tie2 activity [48]	Heterogeneous
	CA1-1	pTie2, pAKT [62]	Heterogeneous
	CA1-2	no Tie2 activity [62]	Heterogeneous
	H3, Tet1A, Icos1-A	pERK, pFAK [21]	Homogeneous
4	C4, AkC4	pERK, pFAK [21]	Homogeneous
	MAT-Ang2	pAKT [48]	Heterogeneous
	MAT-Ang 1	pTie2, pAKT [61]	Heterogeneous
5	Comp-Ang 1	pTie2, pAKT [61,62]	Heterogeneous
	Comp-Ang 2	pTie2, pAKT [48]	Heterogeneous
6	H6	pAKT, pERK, pFAK [21]	Homogeneous
8	H8	pAKT, pERK, pFAK [21]	Homogeneous
12	Tet1 & Tet2	pAKT, pERK, pFAK [21]	Homogeneous
24	O42.1	pAKT, pERK [63]	Homogeneous
60	Icos1 & Icos2	pAKT, pERK, pFAK [21]	Homogeneous
	Cap-Fd	pAKT [64]	Homogeneous
	I52.6	pAKT, pERK [63]	Homogeneous
Variable	Ang 1, Ang 2	pAKT, pTie2, pFAK, pERK [21,42,43,44,48,51,54,55]	Heterogeneous

**Table 2:** De novo-designed proteins demonstrated promising signaling modulation and cellular outcome.

De Novo Binders	Target Receptor	Activity
mb7	FGFR1	Inhibits FGFR1 as a monomer Activates pFGFR1/pERK/calcium release when linked to a hexameric scaffold [80]
IR agonists	insulin	Activate pAKT/pERK and promote cell proliferation in C2C12-IR cells [82]
Neonectin	Integrin α5β1	Inhibits integrin-mediated cell adhesion, angiogenesis, and cell migration [79]
5HCS binder	TGFβRI	Inhibit SMAD signaling [81]

## Data Availability

No new data were created or analyzed in this study.

## Abbreviations

Fd	Ang 1 F-domain
Ang 1-Fc	2 Ang 1 F-domains fused with the antibody Fc domain
CA1-3	CMP-Ang variants, coiled-coil domain of Ang1 replaced with a short, trimeric coiled-coil domain of cartilage matrix protein
GCN4-Ang 1	Ang 1 F-domain fused to coiled-coil domains of GCN4
GCN4-Ang2	Ang 2 F-domain fused to coiled-coil domains of GCN4
CA1-1 and CA1-2	CMP-Ang variants, coiled-coil domain of Ang1 replaced with a short, trimeric coiled-coil domain of cartilage matrix protein
C4	Modified tpr1C4F to form tetramer and presents 4 Ang 1 F-domains
AkC4	Tetramer formed by 4 ankyrin domain repeats presenting 4 Ang 1 F-domain
MAT-Ang 1 or MAT-Ang 2	Matrilin-1 protein fused with Ang 1 or Ang 2 F-domain
Comp-Ang 1 or Com-Ang 2	Cartilage Oligomeric Matrix Protein with Fd of Ang 1 or Ang 2
H3, H6, or H8	Helical bundles conjugated with 3, 6, or 8 Ang 1 F-domains
Tet1 & Tet2	Tetrahedral nanocage 1 or 2 conjugated with 12 Ang 1 F-domains
Tet1A	Trimeric subunit of Tet1 presenting 3 Ang 1 F-domains
O42.1	Octahedral nanocage formed with Ang 1-Fc to present 24 Ang 1 F-domains
Icos1 & Icos2	Icosahedral nanocage 1 or 2 conjugated with 60 Ang 1 F-domains
Icos1-A	Trimeric subunit of Icos 1
Cap-Fd	De novo-designed capsid cage conjugated with 60 Ang 1 F-domains
I52.6	Icosahedral nanocage formed with Ang 1-Fc to present 50 Ang 1 F-domains
